# Current Progress in Particle-Based Systems for Transdermal Vaccine Delivery

**DOI:** 10.3389/fimmu.2020.00266

**Published:** 2020-02-26

**Authors:** Jonas Pielenhofer, Julian Sohl, Maike Windbergs, Peter Langguth, Markus P. Radsak

**Affiliations:** ^1^Biopharmaceutics and Pharmaceutical Technology, Johannes Gutenberg-University, Mainz, Germany; ^2^Third Department of Medicine - Hematology, Oncology, Pneumology, University Medical Center of the Johannes Gutenberg-University, Mainz, Germany; ^3^Institute of Pharmaceutical Technology, Buchmann Institute for Molecular Life Sciences, Goethe-University, Frankfurt, Germany

**Keywords:** needle-free vaccination, transcutaneous immunization, drug delivery, particulate systems, nanoparticles, vaccine particles

## Abstract

Transcutaneous immunization (TCI) via needle-free and non-invasive drug delivery systems is a promising approach for overcoming the current limitations of conventional parenteral vaccination methods. The targeted access to professional antigen-presenting cell (APC) populations within the skin, such as Langerhans cells (LCs), various dermal dendritic cells (dDCs), macrophages, and others makes the skin an ideal vaccination site to specifically shape immune responses as required. The stratum corneum (SC) of the skin is the main penetration barrier that needs to be overcome by the vaccine components in a coordinated way to achieve optimal access to dermal APC populations that induce priming of T-cell or B-cell responses for protective immunity. While there are numerous approaches to penetrating the SC, such as electroporation, sono- or iontophoresis, barrier and ablative methods, jet and powder injectors, and microneedle-mediated transport, we will focus this review on the recent progress made in particle-based systems for TCI. This particular approach delivers vaccine antigens together with adjuvants to perifollicular APCs by diffusion and deposition in hair follicles. Different delivery systems including nanoparticles and lipid-based systems, for example, solid nano-emulsions, and their impact on immune cells and generation of a memory effect are discussed. Moreover, challenges for TCI are addressed, including timely and targeted delivery of antigens and adjuvants to APCs within the skin as well as a deeper understanding of the ill-defined mechanisms leading to the induction of effective memory responses.

## Introduction

The skin is the outer barrier of our body that executes a plethora of essential functions, including maintenance of fluids, regulation of body temperature, sensing of pain, and sheltering from external aggressors. These functions are accomplished by both the unique anatomy and the colonization of the skin by a versatile network of skin-associated immune cells that permanently monitor their tissue environment for invading pathogens. Due to the easy accessibility and the prevalence of a profoundly complex and functionally rich network of immune cells in the skin, the interest in transcutaneous immunization (TCI) approaches has successively increased since the proof-of-concept was made by Glenn and co-workers two decades ago (1). Particularly interesting for vaccination via the skin are the unique, but heterogeneous populations of professional antigen-presenting cells (APC) located in the viable epidermis and the dermis, respectively. Since the initiation of a powerful adaptive immune response requires optimal antigen presentation by professional APCs, the innate cutaneous immune system is of notable interest for vaccination via the skin (2).

Human skin consists of three major layers: Epidermis (subclassified into *stratum corneum* and viable epidermis), dermis, and subcutis. The basal part of the epidermis is populated by a specialized subtype of dendritic cells, named Langerhans cells (LCs). Langerhans cells are uniquely located in the epidermal layer and build up the first line of APCs that encounter skin-invading antigens. A multitude of scientific reports indicate a crucial role for LCs in the induction of CD8^+^ T-cell responses, likely due to their ability to cross-present antigens to naïve or memory CD8^+^ T cells (3). The development and employment of inducible transgenic rodent models (e.g., the Langerin-DTR mouse model) recently challenged the essential need for LCs as antigen-presenting cells in the skin and emphasized the importance of dermal dendritic cells (dDCs) (4, 5).

Dermal DCs represent a highly mixed subset with functional heterogeneity and have been identified as key players in the induction of immune responses both in cutaneous infection and in skin vaccination (6). Based on their developmental origin, surface markers, and function, dDCs can be broadly subdivided in steady-state conditions. The dermis is inhabited by two conventional subtypes of dDCs, both originating from a common bone-marrow-derived Lin^−^ cKit^int^ M-CSFR^+^ Flt3^+^ precursor. The XCR1^+^ cDC1 subtype is functionally specialized in antigen cross-presentation, polarization of T helper cells into the T_H_1 subset, and secretion of IFNγ, which emphasizes its crucial role in acting against intracellular pathogens (7). The CD4^+^CD11b^+^ subset represents a separate DC lineage (“cDC2”) specialized in the presentation of antigen to CD4^+^ T cells and with the unique ability to favor polarization toward T_H_2 or T_H_17 responses, which emphasizes their importance during immune responses to extracellular pathogens. The development of the cDC2 lineage is highly dependent on the transcription factor IRF4 (8). Moreover, it has been shown that the cDC2 lineage is also able to prime CD8^+^ T cells independently (9). However, recently published reviews address the diversity of the cutaneous APC network and facilitate a profound understanding of immunological processes in the skin (2, 10).

**Figure 1:**
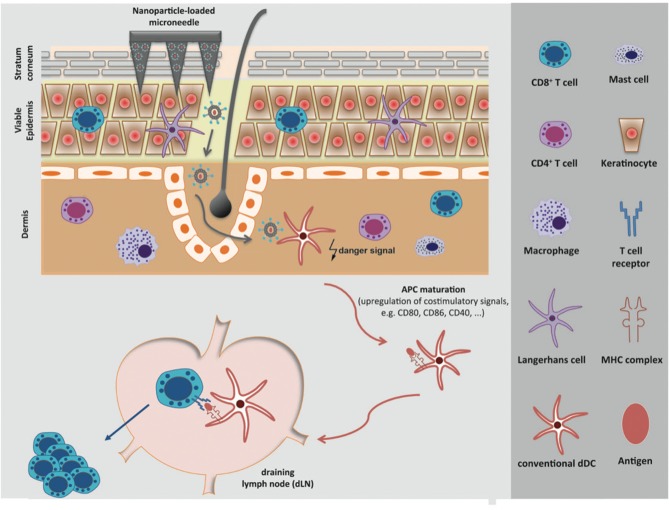
Overcoming the *stratum corneum* with particle-based systems and targeting of the APC network in the skin. Particle-based systems for transdermal vaccine delivery facilitate the targeting of the versatile network of skin-resident antigen-presenting cells (APCs). Activated APCs incorporate an antigen and migrate to the draining lymph node (dLN) where naive T cells are primed, thereby enabling an antigen-specific cellular immune response.

The ideal targeting of cutaneous APC populations by a skin-compatible adjuvant agent appears to be indispensable for the induction of a powerful adaptive immune response and the initiation of immunological memory. Immunological adjuvants are defined as any substances that act to accelerate, prolong, or enhance antigen-specific immune responses when used in combination with a specific vaccine antigen. Adjuvant agents initiate the maturation of cutaneous APCs, promote the migration from the skin to the draining lymph node (dLN) and enable ideal antigen-presentation to naïve T cells by the up-regulation of co-stimulatory molecules on the APC, thereby avoiding the induction of weak or anergic T cells (summarized in Figure 1). Commonly used adjuvant agents in particle-based systems target toll-like receptors (TLRs), nucleotide-binding and oligomerization domain (Nod)-like receptor (NLRs), mannose receptors, and complement receptors. Congruously to the immense importance of adjuvant agents, numerous efforts have been made in this research field, which have been excellently summarized for vaccination in general and especially for particle-based systems (11, 12).

The greatest obstacle preventing ideal targeting of skin-resident APCs by vaccines is the anatomy of the *stratum corneum*. Therefore, a diversity of strategies to overcome the *stratum corneum* has recently been presented (13–16). In this review, we will address progress made with particle-based systems used for transcutaneous immunization (TCI) for optimized antigen and adjuvant delivery. In general, particle-mediated TCI is achieved by either active vaccine delivery enhancing vaccine penetration by compromising the SC or by passive delivery based on passive diffusion of vaccines into the skin. An overview of the particle type, the used delivery method, the infection type and the characterized immune response is presented in Table 1.

**Table 1:** Overview of the particle type used, delivery technology, infection type, antigen and adjuvant used, and induced immune response in some of the presented studies.

	**Particle type and/or delivery technology**	**Infection type/antigen/adjuvant**	**Induced immune response**
Active particulate delivery	Microneedle (MN) skin pretreatment followed by application of soybean phosphatidylcholine/span 80 elastic vesicles (17)	Hepatitis B/ hepatitis B surface antigen (HBsAg)/Cholera toxin (CT)	- Adjuvanted formulations induced significantly higher titers of anti-HBsAg antibodies (IgG, IgG1a, IgG2a) than formulations without CT after MN pretreatment but significantly lower titers compared to intramuscular (IM) immunization
	MN loaded with cationic polylactic-co-glycolic acid-poly-l-lysine/poly-γ-glutamic acid (PLGA-PLL/γPGA) nanoparticles (NP) (18)	Ebola/Ebola DNA vaccine (EboDNA) coated onto the NPs/ no adjuvant	- Comparable antigen-specific IgG-levels for IM, MN immunization with NP or after IM injection of naked EboDNA - Significantly higher IgG1 subtype responses for IM immunization with NP and higher responses for MN immunization with NP compared to IM immunization with naked EboDNA - No significant difference in IgG2a levels between IM/MN immunization with NP and IM injection of naked EboDNA - Highest neutralizing antibody activity against Ebola GP-mediated virus entry for MN-mediated TCI with NP in pseudovirion neutralizind assay
	MN loaded with EV-71 Virus-like particles (VLPs) (19)	Hand-foot-and-mouth-disease/Enterovirus 71 (EV71)/no adjuvant	- Comparable levels of IgG for MN and IM immunized mice but significantly higher IgG responses for MN immunization compared to SC injection of EV71-VLPs - Balanced T_H_1/ T_H_2 response for IM or MN immunization compared to SC immunization - Significantly higher numbers of IFN-γ - and IL-4-secreting cells after immunization with MNs than after IM or SC injection
	Iontophoresis and OVA-loaded liposomes and silver nanoparticles (NPag) (20)	Model antigen Ovalbumin (OVA)/no adjuvant	- Significantly higher IgG1 and IgG2a levels after second immunization with iontophoresis and OVA-liposomes/NPag compared to the negative group - Comparable IgG1-titers after the second immunization with iontophoresis and OVA-NPag-liposomes and subcutaneous injection (SC) - Iontophoretic treatment with OVA-NPag-liposomes resulted in higher levels of activated T CD4 and B CD 19 cells; in contrast, cytotoxic T CD8 expression was not increased - Iontophoresis alone activated the expression of total B lymphocytes
Passive particulate delivery	OVA-loaded chitosan nanoparticles (CS-NP) (21)	Model antigen OVA/adjuvant imiquimod	- CS-NPs with the adjuvant revealed comparable levels of anti-OVA IgG titers to SC injection of an OVA solution, - Significantly higher IgG-levels after topical application of OVA-loaded CS-NPs in comparison to topical application of an OVA solution - Higher survival rate of tumor-bearing mice after TCI with antigen gp100-loaded CS-NP in comparison to gp 100 antigen solution
	Solid in oil dispersions (S/O) carrying MHC-I antigen-binding peptide TRP-2 (22)	Melanoma/MHC-I antigen-binding peptide TRP-2/ resiquimod (R-848)	- Comparable inhibition efficiency of tumor growth for the S/O formulation compared to SC injection of the TRP-2 antigen and to that of administration of pure resiquimod solution - Suggested induction of T-cell responses for S/O-dispersion based on the infiltration of cytotoxic T lymphocytes in tumor tissue - Significant decrease in tumor growth rate in mice vaccinated with S/O containing R-848; three of the five mice in the group had rejected the tumor implantation by day 31
	Solid nanoemulsion carrying nano-dispersed imiquimod with SIINFEKL (23–25)	Model Antigen SIINFEKL/ Imiquimod and, where appropriate, CD40 ligands	- Enhanced primary CD8+ and CD4+ T-cell responses and tumor protection when vaccinated with the solid nanoemulsion (SN) in comparison to the reference formulation, Aldara® - Co-application of the SN with co-stimulatory ligands such as CD40 ligands, promotes specific T-cell responses in the priming and memory phase, strongly enhancing antitumor protection in mice

## Particle-Based Systems for Transcutaneous Vaccination

### Active Delivery Methods

Active delivery of particle-based systems includes technologies that involve the application of various devices to actively enhance skin permeability to vaccines. These methods include transdermal electroporation (26), sonophoresis (27, 28), microneedle patches (13, 29, 30), skin radiofrequency/thermal and laser ablation (31–34), jet or powder injection (15, 35), and iontophoresis. Applying these methods together with vaccines formulated as particulate systems enhances their skin penetration and their recognition of and interaction with APCs (36).

Alongside this intrinsic adjuvanticity of particles, the type and quality of the immune response also alters with changes in the compositions of particles and their physicochemical properties (e.g., size, shape, charge, hydrophobicity). For example, particle size was found to contribute to cross-presentation efficiency, with smaller particles enhancing cross-presentation efficiency (37, 38). Other particulate characteristics participating in vaccination outcome include positive particle charge enhancing cellular internalization via electrostatic attractive forces between particles and negatively charged cell membranes, particle hydrophobicity, and particle shape. However, their influence on immune system activation is not yet fully understood [see (36, 39–41)].

Several studies have been performed to assess the use of active delivery methods together with particle-based systems for TCI, i.e., microneedles (17, 18, 42), iontophoresis (20), jet or powder injection, or mild skin ablation by cyano-acrylate skin surface stripping (CSSS) (43). In some of these studies, the combined approaches for TCI with our without the use of adjuvant(s) were reported to enhance vaccine skin entry, activate the innate immune system, thereby serving as a physical adjuvant, and lead to higher or altered levels of humoral and cellular immune responses compared to subcutaneous (SC) or intramuscular (IM) vaccination with particulate or soluble antigens (19).

However, active delivery of particle-based systems is also associated with some limitations. Although these combined approaches, together with the addition of adjuvants, can enhance vaccine skin penetration and immunogenicity, the induction of strong protective immune response may not be achieved. This can most likely be related to the lack of vaccine immunogenicity, underlining the necessity of optimizing vaccine formulations further to induce strong and protective immune responses against pathogens or for vaccination against cancer. Regarding the active vaccine delivery method itself, their enhancement of skin permeability is often based on skin abrasion, an undesirable feature that considerably increases the risk of pathogen entry and cross infection, which is undesirable in mass vaccination campaigns. However, some of these techniques have good potential for combination with particle-based systems. Microneedles represent widely studied minimally invasive drug delivery systems that are promising for particle-based vaccine delivery into the skin. Another interesting technique is to only “gently” disrupt the stratum corneum via cyanoacrylate skin surface stripping (CSSS). This increases the number of hair follicles available for particulate penetration but, in parallel, activates LCs and promotes amplification of CD8 effector T-cells (44). Nevertheless, development of non-barrier-disruptive methods is desirable.

### Passive Delivery Methods

Passive delivery of particle-based systems includes noninvasive administration of micro- and nanoparticles or lipid-based systems, for example, solid nano-emulsions and vesicles, avoiding skin-compromising methods. Antigen delivery relies on the passive diffusion of the particles through the intact skin by the formation of a concentration gradient, increasing the hydration of the skin by occlusion and transfollicular diffusion (45). In general, these methods are less time-consuming and have a lower risk of secondary infections compared to barrier-disruptive methods. However, the uptake of vaccines can be delayed and/or restrained.

Micro- and nanoparticles (NPs) are attractive antigen and adjuvant carriers for TCI because of their noninvasive delivery of antigens to APCs in the skin (46, 47). Specifically, the use of particles of a size of a few hundred nm is of interest for TCI because they migrate into the hair follicles (HF) without barrier disruption and accumulate there and are only slowly cleared by hair growth or sebum production (46, 48, 49). The absorption into HF is driven by the oscillatory movement of the hair inside the follicle, mechanically enhancing HF flux, a so-called ratchet effect (48–51). Primarily, this absorption depends on the size of the particles, with an optimum of ~600nm corresponding to the thickness of overlapping cuticular hair surface cells (51). Negative surface charge and lipophilic surface properties also have a positive effect on follicular absorption (52).

From an immunological perspective, vaccination via the follicular route using NPs offers a high immunological potential due to the associated APC environment surrounding the hair follicle openings including Langerhans cells (LCs) in the epidermis and dermal Dendritic cells (dDCs) in the dermis (53). While pure antigens lack efficacy in penetrating the skin to reach APCs for effective activation of the innate immune cells (53), NP delivery of antigens and adjuvants facilitates follicular absorption as well as prolonged exposure of antigens to APCs, thereby enhancing the antigenicity through sustained antigen release. Although no translocation of NPs from the hair follicle into the living epidermis without barrier-comprising methods has been reported so far (52), it seems that the amount of antigen delivered into deeper regions of the hair follicle and also the strength of the antigen-specific immune response after follicular penetration of NPs in comparison to antigenic solutions, can be increased (21, 54). For instance, OVA-loaded chitosan NPs (CS-NPs) together with the adjuvant imiquimod induced comparable levels of anti-OVA IgG titers to SC injection of an OVA solution, while induction of IgG-levels after topical application of OVA-loaded CS-NPs achieved significantly higher antibody levels than topical application of an OVA solution (21). In terms of cellular immunity, encapsulation of the antigen gp100 into CS-NP followed by TCI revealed a higher survival rate of tumor-bearing mice after follicular TCI in comparison to a gp 100 antigen solution (21). However, the cellular immune responses were not further characterized in the latter study.

While NP-mediated vaccine delivery profits from higher immunogenicity in comparison to soluble systems, it seems that the addition of adjuvants, such as TLR agonists (e.g., imiquimod), ADP-ribosylating toxins (e.g., cholera toxin) and others, is required for induction of strong humoral and cellular immune responses as shown, e.g., by Mittal and coworkers (55). In their study, only the administration of OVA-loaded CS-polylactic-co-glycolic acid (PLGA)-NPs co-administered with the adjuvant bis-(3′,5′)-cyclic dimeric adenosine monophosphate (c-di-AMP) led to the induction of a balanced T_H_1/T_H_2 response, which is necessary for stimulation of strong humoral and cellular immune responses (55). Moreover, the authors showed that the quality of the immune response by stimulating multifunctional CD4+ and CD8+ T-cells characterized by secretion of various key cytokines, e.g., TNF-α, IL-2, IFN-γ, and other necessary cytokines for protection against different pathogens, could be increased (53, 55).

However, transfollicular vaccination using NPs still has some limitations. The use of chemical solvents and the physical stress while manufacturing jeopardizes antigen and adjuvant stability, thereby reducing the antigenicity of the antigens. A simplified approach for antigen encapsulation into inverse-micellar-sugar-glass particles (IMSG-NP) was shown to lead to a higher encapsulation efficiency, better stability, and enhanced follicular delivery of antigen and adjuvant (54) but resulted in different immunological activation. In this study, high levels of IgG1 antibody titers but no IgG2a-titers as compared to a CS-PLGA-NP containing the same antigen and adjuvant were observed. Also, considerable activation of CD4+-T-cells but little or none of the CD8+-T-cell activation required for cancer vaccination was detected (54).

Newer approaches aim to activate selected APC populations and induce tailored adaptive immunity by conjugation of NPs with DC-directed ligands such as mannose, as DCs express high levels of mannose receptors, and to conjugate NPs with DC-directed antibodies (e.g., anti-Clec9a) (53, 56). Furthermore, the addition of a release trigger to the NP formulation, for instance, by co-application with a protease (52), offers the potential to specifically tailor the release at a certain penetration depth where the desired APC population resides.

Lipid-based systems such as (charged) transfersomes (57), ethosomes, cubomoses, niosomes, solid-in-oil dispersions, and (solid) nanoemulsions are also interesting delivery vehicles for non-barrier disruptive TCI (58, 59).

Transferosomes are elastic liposomes, consisting of phospholipids and edge activators, e.g., surfactants, forming (ultra-)deformable vesicles, which increase the skin permeability to antigens in the presence of a hydration gradient by squeezing through the intercellular regions of the intact SC (59, 60). This gives them superior potential for antigen transport to APCs compared to conventional liposomes (61). However, contradictory results on the effectiveness in enhancing skin permeability exist among different studies. For instance, delivery of the HBs antigen DNA with ultradeformable cationic liposomes revealed superior levels of cellular and humoral immune responses compared to vaccination with conventional liposomes (62), whereas Ding et al. report that TCI with ultradeformable liposomes alone did not improve immunogenicity but required skin pretreatment with microneedles (63). However, surface modification of transferosomes, e.g., by coupling them with DC receptor ligands such as mannose, might compensate for the low vaccine levels caused by limited transfersomal vaccine delivery through enhanced DC vaccine uptake (56).

Ethosomes consist of phospholipid bilayer(s) encapsulating a hydroalcoholic solution with high ethanol content (up to 45%), increasing lipid fluidization of the vesicles and of the skin lipids through interacting with polar lipid head groups in the skin, thereby lowering their melting point (60). In some studies, ethosomes were reported to be superior delivery vehicles compared to conventional liposomes and transfersomes. Rattanapack et al. observed that ethosomes were superior vaccine carriers, as shown by improved antigen skin penetration *in vitro* (64). However, *in vivo* immunization studies were not performed. Zhang et al. compared OVA- and saponin-loaded ethosomes, liposomes, and transfersomes as regards their immunization potency in mice (65). *In vivo* immunization experiments revealed the highest anti-OVA IgG titers for immunization with ethosomes compared with liposomal or transfersomal TCI. However, the type and quality of cellular immune responses were not investigated.

Cubosomes are nanostructured dispersions of the bicontinuous liquid, crystalline phase. While Rattanapak et al. could show that cubosomes enhance antigen skin penetration, it is not clear whether they enhance TCI efficacy, since *in vivo* immunization experiments were not performed (64).

Niosomes are non-ionic surfactant vesicles and have also been investigated for TCI. However, their application for TCI revealed relatively low skin penetration and significantly lower induction of antibody titers compared to TCI with transfersomes (66).

Solid-in-oil (S/O) dispersions comprise oily dispersions of particulate surfactant-peptide complexes, which are made by coating hydrophilic peptides with hydrophobic surfactants. Evaluation of these carrier systems for TCI against cancer with the adjuvant resiquimod and the melanoma MHC-I antigen-binding peptide TRP-2 revealed comparable inhibition efficiency of tumor growth for the S/O formulation compared to injection of the TRP-2 antigen and to that of administration of pure resiquimod without the peptide groups (22).

Nanoemulsions (NE) are heterogeneous systems of two immiscible liquids, oily and aqueous in nature, carrying drugs dispersed in nano-sized droplets with droplet sizes of <500 nm into the skin (67). Their enhanced ability to penetrate skin is due to the physico-chemical modification of vaccines, including the small droplet size, high elasticity, low polydispersity, high zeta-potential, and different NE and emulsifier types (68). Moreover, the addition of occlusive substances and the presence of high amounts of surfactants are further options for enhanced vaccine delivery into the skin. The route of delivery for NE is size-dependent in a similar way to NP, where NE mainly enter the skin via the HFs (69). However, it seems that these systems also deliver vaccines into the skin through the intercellular pathway, even if to a small extent due to their deformable nature. Within the literature, the application of NEs for TCI revealed promising immunogenic potential. For instance, incorporation of outer membrane antigens of *Salmonella enterica* into an NE followed by TCI revealed higher epidermal and transfollicular antigen uptake and resulted in significantly higher IgG antibody titers compared to TCI with ointment formulations (70). Interestingly, encapsulation of the antigen into poly(anhydride) nanoparticles and incorporation into an NE did result in significantly lower antibody titers.

In our research group, we developed nano-dispersed imiquimod formulations together with the synthetic peptide SIINFEKL and compared them for their vaccination potency against the commercially available imiquimod formulation Aldara® (71, 72). Despite the reduced skin flux in *in vitro* experiments of the nano-dispersions compared to Aldara®, this seemed not to affect vaccination potency in *in vivo* experiments, since the solid nanoemulsion formulation (SN) revealed strongly enhanced primary CD8+ and CD4+ T-cell responses compared to Aldara®. This underlines that prolonged exposure to adjuvant and antigen is necessary for DC activation. In addition, application of the SN together with co-stimulatory signals, e.g., CD 40 ligands, revealed robust memory formation and enhanced tumor protection (23–25).

## Conclusion

In conclusion, particle-based systems have great potential for transcutaneous vaccine delivery via the skin. Their active skin delivery can enhance vaccine immunogenicity due to achieving better particulate APC recognition and activation compared to soluble antigens. In addition, the type and quality of immune responses can be altered by varying the particle characteristics, such as size charge, hydrophobicity, material, and shape, as they participate in vaccination outcome (cross-presentation efficiency, cellular internalization, innate immune system activation). Particulate vaccine delivery with passive methods enhances vaccine entry and activation of APCs predominantly through the entrance and accumulation of particles in the HF openings, where they expose APCs to antigens for a prolonged time period, which can promote the induction of humoral and cellular immune responses. In contrast, penetration of particles through the intact stratum corneum with passive delivery methods seems to play a subordinate role.

However, while some progress has been made in particle-based transcutaneous vaccination via the skin (active or passive), it is not yet completely clear how to formulate the particulate vaccine formulations to deliver vaccines into the skin effectively and, in parallel, sufficiently activate the immune system. A deeper understanding of the effects of particle characteristics (e.g., size, shape, material, hydrophobicity) and other immunization parameters (e.g., skin condition, age, administration site) on immunization outcome will allow accurate engineering of vaccine formulations to enhance skin penetration and polarize CD4+ T-cell differentiation to achieve tailored immunity. In perspective, such vaccination approaches should lead to increased vaccination efficacy in persistent infections and cancer and to patient compliance, overcoming many of the current limitations of standard vaccinations.

## Author Contributions

JP and JS performed literature research and participated in writing the manuscript. MW made important suggestions to the concept and participated in writing the manuscript. PL and MR designed the concept of the study and participated in writing the manuscript.

### Conflict of Interest

The authors declare that the research was conducted in the absence of any commercial or financial relationships that could be construed as a potential conflict of interest.
